# Percy on Reduction of Dislocation of the Hip, by Manipulation

**Published:** 1860-07

**Authors:** 


### PERCY ON REDUCTION OF DISLOCATION OF THE HIP BY MANIPULATION.

Prof. Hamilton, in his recent work on fractures cites a number of authorities going to prove the use of this method of treating dislocation of the hip, at various periods, and in different countries. He seems to have overlooked so high an authority as Baron Percy, who, about the year 1793, (year 3d of the French Republic,) in reply to what were called "*Questions Epuratoirs*," purifying questions, proposed by the "*Commission de Sante*," to all the army and hospital surgeons, to test their capacity and qualifications for the situations which they they occupied respectively. Percy, was, at the time, surgeon-in-chief of the army of the Moselle, but was obliged, like the rest, to bow to the dreaded power of the Convention. The *Responses du Citoyen Percy* are found in the appendix to his work, entitled, *Pyrotechnie Chirurgical Practique*, or the art of applying fire in surgery.

"The reign of lacqs, blocks, and machines, is past, for good surgery. These violent means restrain the action of the muscles too much; prevent them from relaxing at the proper time, when it is only necessary to direct the bone towards its cavity; lay out upon them unequal forces; expose them as well as well as the ligament to fatal tearing. They take away from the operator the power of giving to the forces that extensive change of direction which the particular position of the luxated bone may require," etc., p. 19.

"The practice of the ancients was rarely sufficient to reduce luxations of the thigh. The extensions, which were excessive, might well draw the head of the femur near it cavity but this is not enough." "It is impossible to replace it if the thigh is not free from all restraint. Thus the operators who lengthen the member with cords, pullies, etc., must often fail." p. 22.

"It is certain, at the present day, that but very little force is required to reduce a dislocation of the hip and that in no case ought lacqs and machines to be resorted to. The method of proceeding is as follows: The patient being laid upon the back, carry the dislocated member inward and place it beside the sound one. An aid supports the knee which in this luxation is always flexed, and extends it, which puts the muscles in a state of parallelism calculated to produce a full extension of all and thus prevent resistance. The operator then seizes the foot with the two hands placed one above and the other below, without raising it; he draws it toward him in a direct line, gently at first so as to make the muscles yield more easily, afterward more strongly so as to bring the head of the bone opposite its natural place; he then gives it some lateral movements which complete the reduction. p. 22.

